# CoQUAD: a COVID-19 question answering dataset system, facilitating research, benchmarking, and practice

**DOI:** 10.1186/s12859-022-04751-6

**Published:** 2022-06-02

**Authors:** Shaina Raza, Brian Schwartz, Laura C. Rosella

**Affiliations:** 1 Public Health Ontario (PHO), Toronto, ON Canada; 2 Dalla Lana School of Public Health, University of Toronto, Toronto, ON Canada

**Keywords:** COVID-19, Transformer model, Question answering system, Pipeline, CORD-19, LitCOVID, Long-COVID, Post-COVID-19

## Abstract

**Background:**

Due to the growing amount of COVID-19 research literature, medical experts, clinical scientists, and researchers frequently struggle to stay up to date on the most recent findings. There is a pressing need to assist researchers and practitioners in mining and responding to COVID-19-related questions on time.

**Methods:**

This paper introduces CoQUAD, a question-answering system that can extract answers related to COVID-19 questions in an efficient manner. There are two datasets provided in this work: a reference-standard dataset built using the CORD-19 and LitCOVID initiatives, and a gold-standard dataset prepared by the experts from a public health domain. The CoQUAD has a Retriever component trained on the BM25 algorithm that searches the reference-standard dataset for relevant documents based on a question related to COVID-19. CoQUAD also has a Reader component that consists of a Transformer-based model, namely MPNet, which is used to read the paragraphs and find the answers related to a question from the retrieved documents. In comparison to previous works, the proposed CoQUAD system can answer questions related to early, mid, and post-COVID-19 topics.

**Results:**

Extensive experiments on CoQUAD Retriever and Reader modules show that CoQUAD can provide effective and relevant answers to any COVID-19-related questions posed in natural language, with a higher level of accuracy. When compared to state-of-the-art baselines, CoQUAD outperforms the previous models, achieving an exact match ratio score of 77.50% and an F1 score of 77.10%.

**Conclusion:**

CoQUAD is a question-answering system that mines COVID-19 literature using natural language processing techniques to help the research community find the most recent findings and answer any related questions.

**Supplementary Information:**

The online version contains supplementary material available at 10.1186/s12859-022-04751-6.

## Background

Coronavirus disease (COVID-19) is an infectious disease caused by the SARS-CoV-2 virus [1]. The coronavirus was named the severe acute respiratory syndrome coronavirus-2 (SARS-CoV-2, 2019-nCoV) due to its high homology (80%) to SARS-CoV, which caused Acute Respiratory Distress Syndrome (ARDS) and high mortality during 2002–2003 [2]. The World Health Organization (WHO) declared COVID-19 a pandemic on 11th March 2020 [3]. COVID-19 has been reported in approximately 200 countries and territories [1]. As of 24^th^ February 2022, over 430 million COVID-19 cases have been reported worldwide, resulting in over 5.94 million deaths.^1^

The COVID-19 pandemic has also resulted in a significant increase in mental health issues such as depression, post-traumatic stress disorder, and suicide as a result of social distancing measures and state-by-state quarantine [4]. Following the quarantine period, individuals become isolated and stressed, resulting in long-term psychological consequences [5, 6]. The post-COVID-19 condition [7], also known as long-COVID, is a newly documented condition that is recognized by the Public Health Agency of Canada (PHAC)^2^ and WHO [8]. The post-COVID-19 condition usually manifests four to twelve weeks after the initial infection [9]. It is a multisystem disease including multiple organ systems (pulmonary, cardiovascular, neurological, musculoskeletal), and mental health conditions (depression, mood or anxiety disorders, dementia) [6]. To date, the short and long-term effects of the post-COVID-19 condition are still largely unknown [6].

The worldwide response to COVID-19 has resulted in a rapid increase in the number of scientific publications about SARS-CoV-2 infection, health and societal impacts [10]. Scientific information is disseminated through a variety of print and digital channels, including formal publication sources such as clinical trials, systematic reviews, preprints, and academic publications [11]. In comparison to printed media, the amount of digital evidence [12] is growing exponentially, necessitating the development of additional research tools to manage it.

Now that a wealth of material on COVID-19 is available, a significant challenge is locating relevant and reliable information within the massive literature on time. Simultaneously, there is an increase in demand for accurate and reliable COVID-19 information from both the medical research community and the public. Concerns about post-COVID-19 health care needs are also emerging [13] and the evidence is rapidly evolving. According to the Ontario Science Advisory Table [14], the number of people with or at risk of developing these post-COVID-19 conditions is a key concern.

High-priority scientific questions such as: “What are COVID-19 risk factors?”, “What is the post-COVID-19 condition?", “What is an effective treatment for post COVID- 19 syndrome?” and such, needs to be addressed. People who are at the front of the field, like doctors, and researchers, are often limited by time constraints to search through a huge amount of scientific literature to find answers to these important questions. This necessitates the development of a question answering system to help researchers and practitioners extract key information and contexts from published data.

A question answering system (QA) system [15] is a branch of computer science that falls under the categories of information retrieval (IR) and natural language processing (NLP). It is concerned with developing systems that automatically respond to questions posed by humans in natural language. A QA system can help with COVID-19 research and can answer questions related to COVID-19.

Online communities, forums, and social media can also be used to search for relevant answers or to post questions and receive responses from other participants. However, these forums are largely unregulated, so the credibility of information is a key issue. Scoping reviews and systematic reviews are also other approaches to evidence synthesis, with the former focusing on a broad research question and the latter summarizing the best available research on a specific question [16]. It is, however, often difficult for health practitioners and researchers to find immediate answers to many of the real-world questions they encounter. Furthermore, by the time these reviews are completed, a significant amount of new information has already been published in the literature.

Considering the above-mentioned obstacles (sifting through an enormous amount of literature, processing of information on time, the credibility of information), we propose a QA system that can significantly help scientists in their efforts to mine COVID-19 information on time. Our proposed approach, **CO**VID-19 **Q**uestion-**A**nswering **D**ataset (CoQUAD) system is enhanced by Artificial Intelligence (AI) and deep neural networks. Our QA strategy is based on an extractive QA system [17]. Extractive QA is the process of looking through a large collection of documents to extract a concise snippet to answer a question [18].

We provide two datasets in this research: a reference-standard dataset; and a gold-standard dataset. A ‘reference-standard’ [19] generally refers to the collection and compilation of primary and secondary sources of data that can be re-used and cited for various purposes, such as for the biomedical dataset retrieval [20]. The sources of such data are usually from the standard organisations (such as Allen AI, NIST, TREC, and so). The term “reference-standard” dataset is quite often used in this work to refer to the creation of a dataset using publicly available COVID-19 initiatives (CORD-19 and LitCOVID).

The second dataset that we provide in this research is a gold-standard dataset. The term “gold-standard dataset” refers to a dataset that has been manually prepared and annotated by experts [21]. We frequently use the term “gold-standard”, in this work, to refer to our manually annotated COVID-19 corpus used for the QA task. Our objective behind these datasets is two-fold: to build a COVID-19 QA system using a reference-standard dataset, and to evaluate the quality of our QA system using our gold-standard dataset.

There are some COVID-19 QA systems [22–25] that have been proposed in the past; however, many of these works focus (appropriately) on the early COVID-19 period, with an emphasis on disease diagnosis and management, and little coverage of the post-COVID-19 consequences. Recent issues, such as vaccinations, variants of COVID-19, and long-COVID are not represented in these systems. We address this knowledge gap by supplementing our QA system with data from these topics (early, mid-and long-COVID), which we claim to be our contribution.

In this paper, we address the following Research Questions (RQ): (RQ1) How to construct a dataset to find evidence from scientific literature?, (RQ2) How to find the answer(s) to a given question from a large set of documents?. This study focuses on COVID-19, the proposed architecture applies to a wide variety of QA tasks across different domains (healthcare, health science, biomedical, social science or any sub-domain). We summarize our contributions as.

- We develop CoQUAD, a QA system that consists of two datasets (a reference-standard and a gold-standard dataset) and two pipelines (a data processing pipeline and a QA pipeline), to assist researchers, practitioners, and clinical experts in responding to any COVID-19 questions posed in natural language.
- We prepare a reference-standard dataset by obtaining scientific articles through the Coronavirus Open Research Dataset Challenge (CORD-19) [26] and LitCOVID [27] initiatives, both of which contain up-to-date scientific information about COVID-19. This dataset covers a wide range of topics, such as epidemiology, public health, equity, clinical care, vaccine, impacts and post-COVID-19.
- We develop an extractive QA pipeline in CoQUAD that retrieves articles from the reference-standard dataset and then extracts answers from the retrieved articles to address COVID-19 questions. This approach is based on a Transformer-based [28] architecture, which is the latest deep neural network model.
- We prepare a gold-standard dataset consisting of a set of 150 question-answers pairs. This dataset is prepared manually by scientists working in the public health domain. Experts carefully examine these articles to determine their trustworthiness, value, and relevance in the COVID-19 context; they developed the questions after reading these articles and put their answers for each question. We prepare and make this dataset available in the Stanford Question Answering Dataset (SQuAD) format (a prototypical standard for the QA systems) [29].
- We fine-tune MPNet [30] (a Transformer-based model) on our gold-standard dataset and use it inside the QA pipeline to enhance its readability. We also evaluate the performance of our QA system using this gold-standard dataset (to evaluate a QA system, a carefully constructed dataset in SQuAD format is required [31]).

Throughout the paper, we use the term ‘CoQUAD system’ to refer to our full architecture and ‘CoQUAD dataset’ to refer to our gold-standard dataset.

The rest of the paper is organized as follows: “Previous work” section is the previous work, “Data collection” section is the data collection, “Methods” section is about the methods and “Experimental setup” section is the experimental setup, “Results” section discusses the results. “Discussion” section is the discussion and “Conclusion” section is the conclusion.

## Previous work

Given the rapid growth of information about COVID-19, the research community and medical services experts are challenged to receive up-to-date and useful information about COVID-19 on time. COVID-19 research is becoming more available through peer-reviewed scholarly publications. There is a plethora of literature available within a two-year timeframe in response to COVID-19. Broadly, this literature can be divided into the following COVID-19 topics [26, 27], which are epidemiology (study of incidence, distribution, and possible control of diseases in populations); genomics (study of the genetic structures of SARS-CoV-2); disease trajectory; drug discovery; early detection and diagnosis; disease progression and management; risk stratification and hospital resource management; and post-COVID-19 condition The growing need for scientific research has made it a difficult and time-consuming task to sift through such a large amount of data. Research tools like QA Systems [15] have been developed in response to this challenge.

### Question-answering (QA) systems

A QA system is a branch of computer science that combines AI, IR and NLP to create systems that respond automatically to questions posed by humans in natural language [15]. Question types include fact, list, definition, how, why, hypothetical, semantically constrained, and cross-lingual [15, 32]. In general, there are two types of QA systems: closed domain and open domain. The closed-domain QA system [33] is concerned with questions that pertain to a single domain (for example, medicine) and can use domain-specific knowledge to answer questions on specific topics such as Alzheimer's disease or cancer disease. Closed-domain QA typically gives an answer that is a text span from the text. Open-domain QA systems [34] are based on a large, unrestricted domain and are capable of responding to a large number of questions. They can also generate new responses based on the text provided. In this work, we concentrate on the closed-domain QA system, our domain is COVID-19.

Typically, a QA system is concerned with providing precise answers to questions posed in natural language [35]. The task of a QA can be reformulated as a machine learning task, in which systems must extract a precise answer from a paragraph given a question and a paragraph. This is also known as Extractive QA [36]. A task like this can be useful either in the open-domain or the closed- domain.

### COVID-19 related datasets

Several COVID-19 datasets [37] based on scientific literature are made available in the last few months. CORD-19 [26] is an open-access repository of tens of thousands of research publications on COVID-19, SARS-CoV-2, and related coronaviruses for use by the global academic community. CORD-19 Challenge [26] is a challenge offered by the CORD-19 dataset that has called on AI specialists to assist the medical community in generating a variety of data science, advanced analytics, deep neural networks and machine learning models. These tasks range from text summarization [38], and document search [39] to QA systems [22, 23, 25]. LitCovid [27], is another comprehensive resource that provides centralized access to over 228 k PubMed publications relevant to COVID-19. Both the CORD-19 and LitCOVID can be used as reference-standard datasets. The reference-standard dataset commonly refers to benchmarking data from physical science to a technical community that has been evaluated and compiled for ease of use [20, 40].

Other than CORD-19 and LitCOVID, there are a few gold-standard datasets also available. A *gold-standard* [21] dataset has been collected and manually annotated by subject-matter experts. It is a very costly activity in terms of time and effort. The gold labels are the annotations of the highest quality provided by skilled annotators. COVID-QA [29] and CovidQA [25] are also two gold-standard datasets that contain QA pairs (~ 2000 QA pairs for COVID-QA and 124 QA pairs for CovidQA), which have been annotated by volunteer medical specialists.

COVIDRead [24] is another gold-standard dataset that comprises over 1 K manually annotated QA. All these COVID QA datasets are usually made available in the SQuAD^3^ format. The original SQuAD is a crowd-sourced QA dataset with questions on Wikipedia articles and answers from the corresponding reading passage (contexts) or the questions are unanswerable. The SQuAD has evolved into the prototypical QA dataset, shedding light on the recent surge in NLP Language Modeling [41, 42] tasks.

### Transfer learning

Transfer learning is a machine learning technique in which a model trained on one large task is adapted to a second, typically smaller, related task [43] and the Transformer models are built on transfer learning and neural attention techniques [28]. Tang et al. [25] fine-tune a Transformer model on their dataset CovidQA to retrieve the relevant documents according to the questions being posed. Oniani and Wang [44] have fine-tuned GPT-2 [42], a Transformer-based model, on the CORD-19 corpus for the QA task. Möller et al., [29] use the RoBERTa [45], a Transformer, and fine-tune it on their gold-standard data, COVID-QA. So, all these models are fine-tuned on some gold-standard datasets that are in the SQuAD format.

Esteva et al. [39] have developed a COVID-19 (CO-Search) search engine that can estimate the document relevance for each query and a ranker module to rank the answers. This model was evaluated in the TREC-COVID challenge [46]. The TREC-COVID^4^ challenge is a test collection based on the CORD-19 dataset and the challenge is to find answers to COVID-19 questions, while also building infrastructure to improve future research.

All these systems (mentioned above) perform well for QA tasks, but the COVID-19 dataset is quite outdated in these systems (COVID-QA dates back to May 2020, while CO-Search uses the CORD-19 July 2020 version). As a result, these models may not provide answers to questions related to the long- COVID-19 data.

In this paper, we propose a QA system based on the most up-to-date COVID-19 literature. Our QA system is a closed-domain, extractive that can answer questions related to COVID-19, with particular emphasis on the post-COVID-19 syndrome.

## Data collection

We prepare two datasets in this study, which are: (1) a reference-standard dataset and (2) a gold-standard dataset.

### Reference-standard dataset

A reference-standard dataset is often defined by standards organizations and compiled to create a benchmark for research [40]. We use two free resources of scholarly articles: CORD-19 and LitCOVID, to prepare our reference-standard dataset.

*Covid-19 Open Research Dataset (CORD-19)* [26] is a public dataset of academic articles about COVID-19 and related research. CORD-19 is hosted by the Allen Institute in collaboration with The White House Office of Science and Technology Policy (OSTP), the National Library of Medicine (NLM), the Chan Zuckerburg Initiative (CZI), Microsoft Research, and the Kaggle group. The dataset was first released on March 16, 2020, and is updated on daily basis. The dataset consists of publications from PubMed Central, Medline, and preprints from arXiv, bioRxiv, and medRxiv [37] and reports from WHO COVID-19 Database [47].

*LitCovid* [27] is a curated literature hub providing centralized access to over 200 k (and growing) relevant PubMed articles. The articles are updated daily and are divided into sections such as general information, transmission, diagnosis, treatment, prevention, case report, and epidemic forecasting.

*Difference between CORD-19 and LitCOVID*: The difference between CORD-19 and LitCOVID is that CORD-19 focuses on COVID-19 literature more widely, encompassing some portion for other coronaviruses (e.g. SARS and MERS), whereas LitCOVID focuses on tracking COVID-19 publications. LitCOVID contains only articles from PubMed and does not contain any pre-prints (CORD-19 has pre-prints).

#### Construction of the reference-standard dataset

We create a bash script that downloads the most recent versions of CORD-19 and LitCOVID releases from their respective FTP servers.

**Fig. 1:**
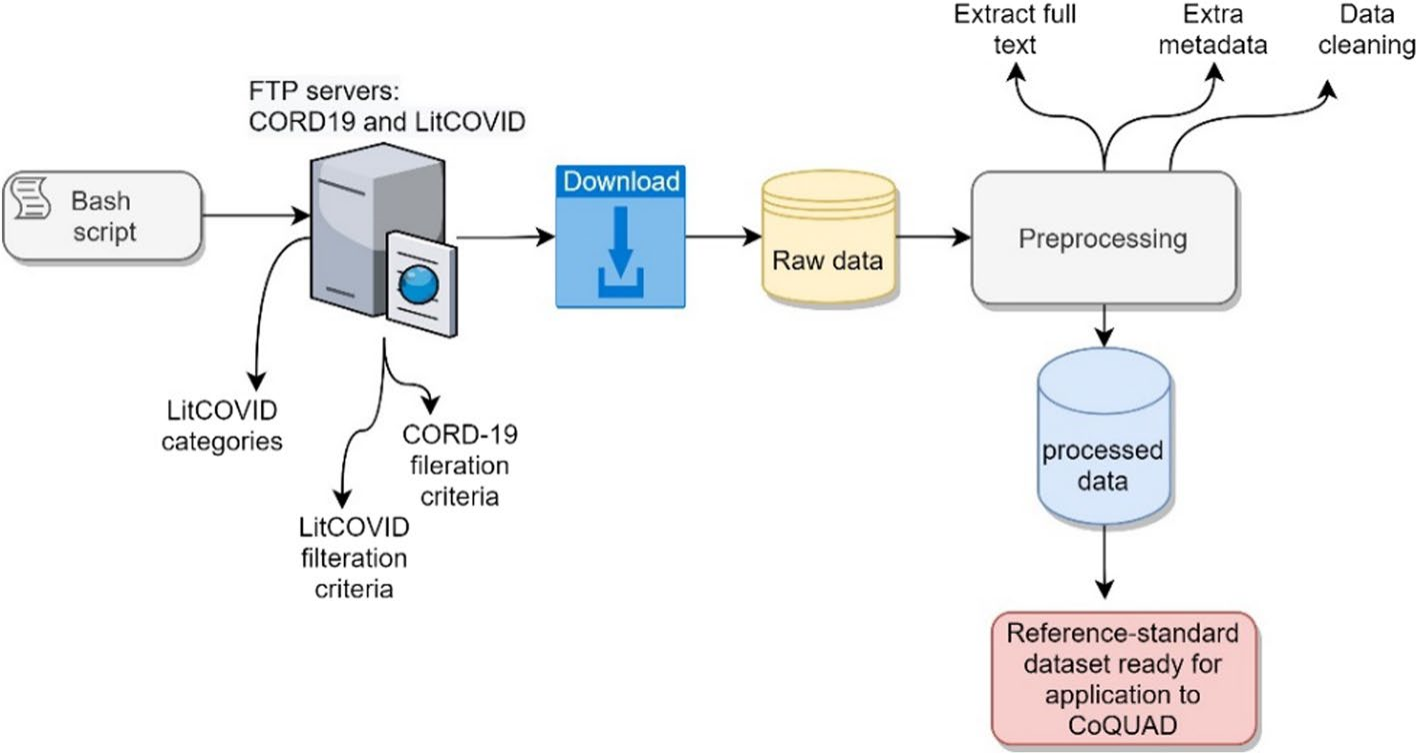
Construction of reference-standard dataset

We use the latest release of CORD-19 from here.^5^ We also obtain the most up-to-date release^6^ for the LitCOVID, both releases were made available by the end of December 2021. To process and store the data from both repositories, we write a Python programme. For the CORD-19, we obtain full articles, PMIDs, and labels. We also process the CORD-19 document embeddings to perform an exploratory data analysis. We obtain the LitCOVID data and preprocess it for full text and metadata. We then parse the data further by dividing longer paragraphs into smaller sections and preparing separate dataframes for each field (PMID, authors, text, abstract, title, DOI). We keep both the processed and raw versions of the data in a directory and use the processed data to construct our reference-standard database, as shown in Fig. 1.

#### Inclusion, exclusion, and filtration criteria

*Inclusion criteria*:

1. We include only the published literature related to COVID-19 in English between 20^th^ March 2020 and 31^st^ December 2021, including guidance/guidelines, reviews, clinical studies, basic research and epidemiological studies.
2. We include the long-COVID collection in addition to early and mid-COVID-19 literature. Long-COVID literature began publishing by the end of July 2021. This inclusion criterion distinguishes our work from previous work in the same field.
3. We only include those papers from CORD-19 that are sourced from PubMed Central (PMC).^7^ LitCOVID papers already provide only PMC articles. So we have PMC collection from both sources.
4. CORD-19 consists of papers in two formats: Portable Document Format (PDF) and Extensible Markup Language (XML) format. We include only the PDFs + PMC articles that provide the full text, abstract and metadata (title, DOI, etc.) for each article. The XML collection does not provide abstracts of the papers [37], so we exclude them.
5. LitCOVID provides data in XML and JavaScript Object Notation (JSON) format,^8^ we include the JSON collection from LitCOVID.

*Exclusion criteria*

1. We exclude papers that were not published in scientific journals, such as pre-prints.
2. We exclude those manuscripts from CORD-19 that are published before 2020 (CORD-19 has some collections before the pandemic [48]).
3. The full LitCOVID dataset consists of more than 228 K articles; we exclude those PMC articles from LitCOVID that are replicating with the CORD-19 dataset.

We merge the articles from both datasets (CORD-19 and LitCOVID) and remove the duplicates using PubMed unique identifier (PMID). A PMID is a unique integer value assigned to each PubMed^9^ record. After all these above-mentioned filtrations (PMC articles + PDFs + removing duplicates + timeline of 2020–2021), we obtain 7978 unique papers from CORD-19 and 9877 articles from LitCOVID. We convert and compile all the data from both sources (CORD-19 and LitCOVID) in JSON format. We parse and convert the JSON formats of these articles and generate a final output in a Comma-Separated Values (CSV) format with main attributes like ‘PMID’, ‘title’, ‘paragraphs’, ‘URL’, ‘publication date’, “DOI”. We also specify the complete text of the research articles (in paragraphs) in the final dataset. The details of the dataset that we use here are given in Table 1. We also perform an exploratory analysis on both datasets and show in Additional file 1: “Appendix A”: *Exploratory analysis*.

**Table 1:** General details of the datasets used in this work

	Total articles	Articles used in this work	Timeline of articles	Files
CORD-19	~ 1,450,000 articles in all formats (PDF, XML)	7978 (only PMC articles)	March 2020 till December 2021	It consists of following files^10^: (1) document embeddings for each paper; (2) collection of JSON files with full text of CORD-19 papers, (3) metadata for all papers, ‘PMID’, ‘title’, ‘paragraphs’, ‘URL’, ‘publication date’, 'DOI'.
LitCOVID	~ 207,630	9877 (PMC articles)	April 2020 till December 2021	The dataset consists of full articles text provided in JSON and XML format. We get the full texts and metdata.

*Both these datasets are updated periodically on COVID-19 articles, we use the data December 2021, which were the latest checkpoints available by 31-December-2021

^10^https://github.com/allenai/cord19

### Gold-standard dataset

A gold-standard dataset is prepared and annotated by experts in the field [49]. A group of experts from the public health domain carefully chose COVID-19 articles on the topics: general information; transmission; diagnosis; prevention; equity; vaccines; and post-COVID. We used a web-based annotation tool^10^ provided by deepset.ai where the annotators (experts in our team) mark the text as answers (gold labels) and formulate corresponding scientific questions.

The gold label is a ground truth value [50], which is an ideal predicted result based on humanly verifiable observation. In this work, it refers to an answer provided by an expert in response to a potential question. This gold-standard dataset is prepared according to the SQuAD 2.0 [31] format, which is a prototypical standard to annotate question-answering pairs. We name and refer to our gold standard dataset as CoQUAD – **CO**VID-19 **Q**uestion **A**nswering **D**ataset.

We answer our first research question here: “How to construct a dataset to find evidence from scientific literature?” by constructing these two datasets (a reference-standard dataset and a gold-standard dataset) from the COVID-19 scientific literature.

## Methods

### Problem definition

This research aims to aid clinical experts, practitioners, scientists, and the research community in obtaining answers to COVID-19 questions. Formally, the problem studied in this paper can be defined as:

“Given a question $q$ and a passage of text $p$, the goal is to find an answer span $a$ from the text.”

*Input:* A question and a passage (context).

- A question *q* is represented by a sequence of tokens: $q = \left\{ {q_{0} ,q_{1} , \ldots ,q_{n} } \right\}$.
- A passage *p* of text is represented by a sequence of tokens: $p = \left\{ {p_{0} ,p_{1} , \ldots ,p_{m} } \right\}$.

Output: an answer.

An answer is represented by* a* sequence of continuous tokens as $a = \left\{ {a_{start} , \ldots ,a_{end} } \right\}$*.,* where $a_{start}$ and $a_{end}$ respectively represent the start and end position of the answer span within *p.* Here*, a* is a subsequence in* p* and $a_{start}$ and $a_{end}$ represents the answer boundary inside *pTask*: The task of CoQUAD system is to learn a predictor function *F*, which maps the question *q* and a paragraph *p* to an answer *a,* as shown in Eq. 1:

1 $$\begin{array}{*{20}c} {F\left( {q,p} \right) \to a} \\ \end{array}$$

**Fig. 2:**
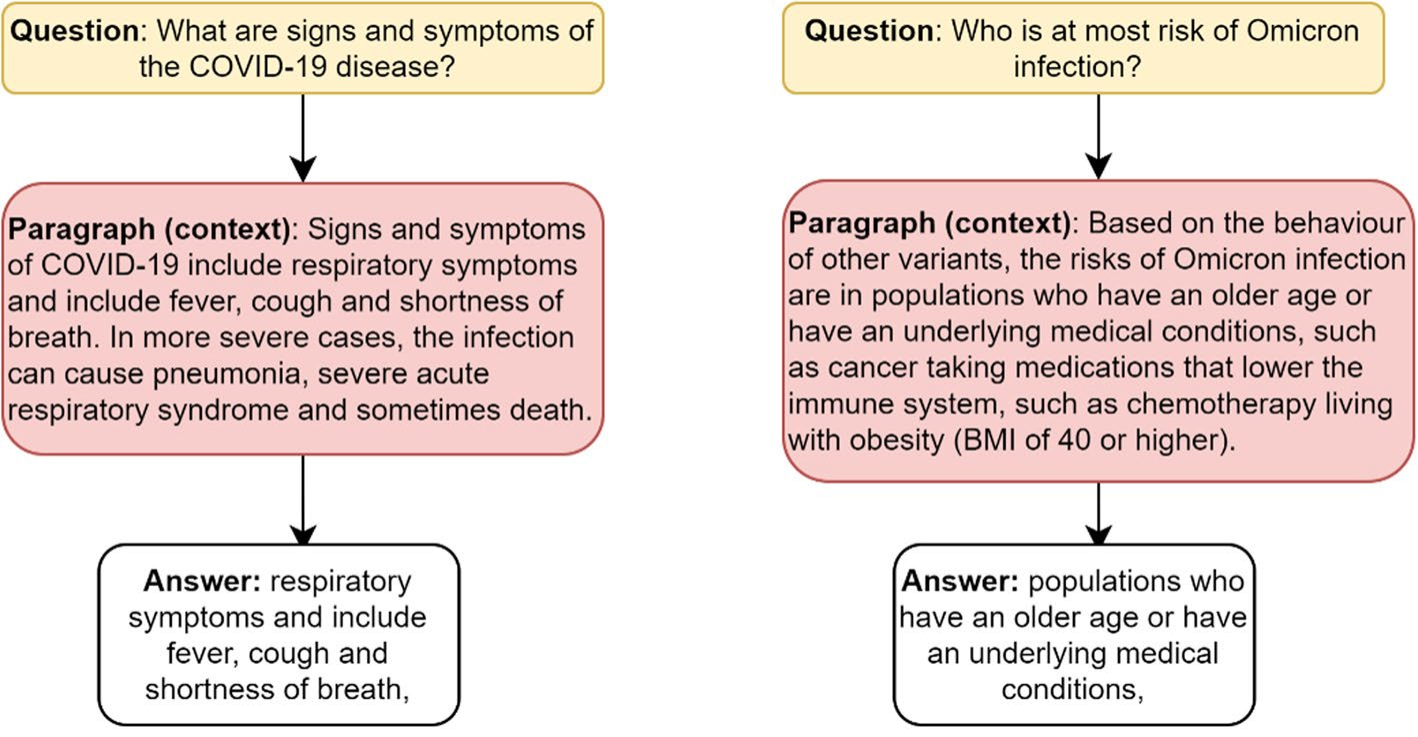
Example of an extractive QA system composed of a question, context and answer

In this work, we develop an extractive QA system for the COVID-19 domain. The task of this QA system is to extract an answer from a text given a question. As illustrated in Fig. 2, extractive QA takes a question and generates an answer based on its context. In contrast to other extractive QA systems, which require explicit context to provide an answer, CoQUAD can provide an answer without explicit context. CoQUAD determines the context for the question, answer, and text of the scientific document based on their semantic similarity.

### CoQUAD framework and its workflow

**Fig. 3:**
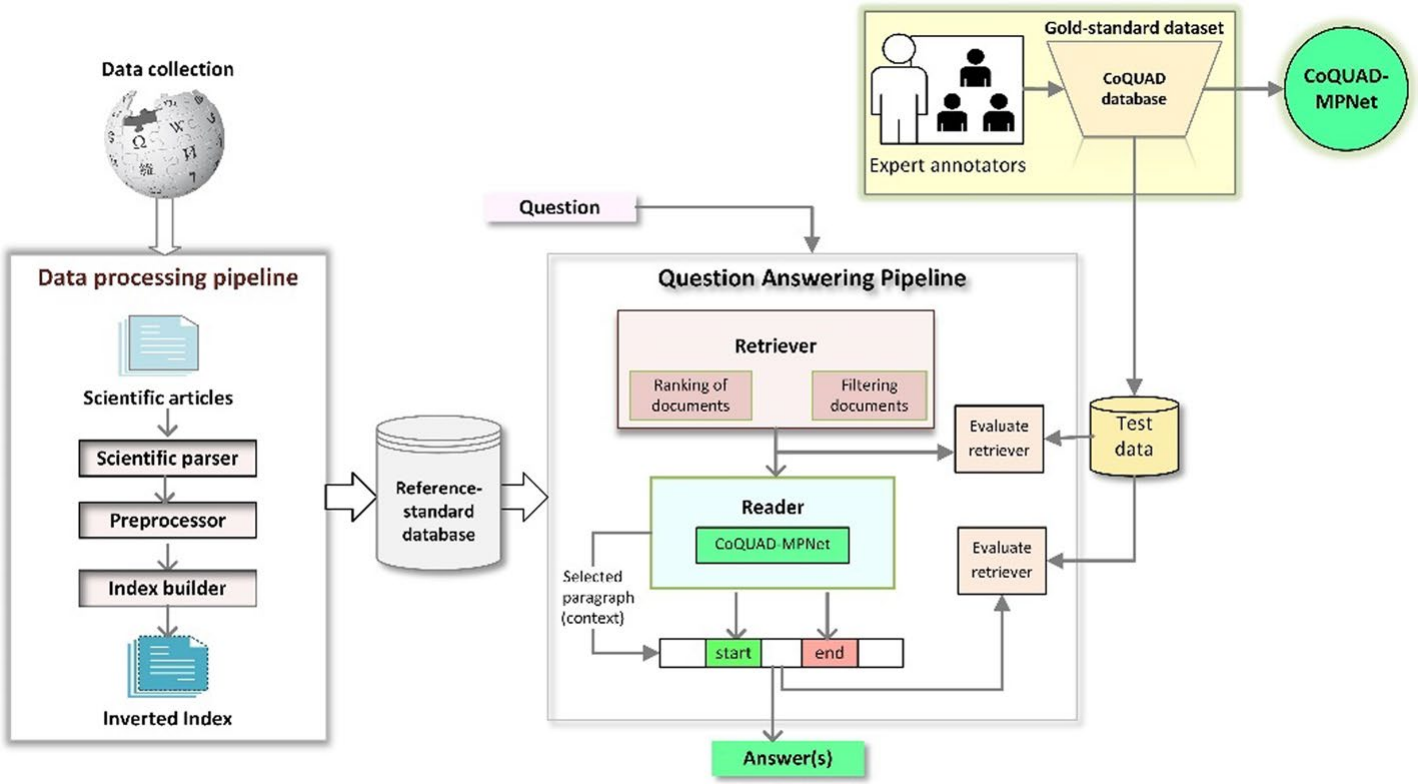
CoQUAD system architecture

We propose an end-to-end framework for our CoQUAD system and show it in Fig. 3. The CoQUAD framework comprises two pipelines: a data processing pipeline and a question answering (QA) pipeline. Each pipeline is made up of core components, called nodes. We also have a data collection phase, a dataset, and an evaluation phase in this framework. Next, we discuss this framework in detail.

The first phase of the CoQUAD framework is data collection. We gather the data from the CORD-19 and LitCOVID repositories and prepare a reference-standard dataset. The details of our data collection strategy are given in “Data collection” section.

#### Data processing pipeline

We develop the data preprocessing pipeline to process and prepare the data for the QA pipeline. The input to the data preprocessing pipeline is a collection of scientific articles and the output is the collection of documents that are parsed, pre-processed, and indexed. Our data processing pipeline consists of three sequential stages: (1) scientific parsing; (2) pre-processing and (3) index building.

*Scientific parsing* The scientific parsing stage is handled by the scientific parser node. The input to the scientific parser is the collection of PubMed articles from our reference-standard dataset. The scientific parser parses these articles into a structured form (title, authors, abstract, paragraphs, bibliography) that is readable and interpretable by any machine learning model. We use Apache Tika^11^ for parsing the text from these articles. The output from the scientific parser is the collection of articles that are parsed and ready to be input to the next stage (i.e., preprocessing) in the data processing pipeline.

*Preprocessing* The second stage in the data processing pipeline is the preprocessing stage, which is handled by the preprocessor node. The input to the preprocessor is the collection of already parsed articles from the scientific parser. The preprocessor performs the tasks, such as cleaning text, removing whitespace, and splitting lengthy articles into multiple smaller and manageable units. Cleaning and splitting texts are critical steps that have a direct impact on the speed and accuracy of QA searches in the later phases. Particularly, splitting lengthy texts is critical for achieving high query performance [51]. The preprocessor also prepares all articles as having a consistent dictionary format that is to be utilized by later nodes in the QA pipeline to make the most of the data. The output from the preprocessor is the collection of articles that are ready to be input to the next stage (i.e., index building).

*Index building* The last stage in the data processing pipeline is the index building, which is handled by the index builder node. The input to the index builder is a collection of preprocessed articles from the preprocessor.

Indexing is the process of transforming items (e.g., documents, research papers, web pages) into a searchable data structure [52]. One might think of an index as a book index, consisting of a list of words and their corresponding page references that direct readers to the locations of various topics within a book. The index provides a link between the terms and the documents that contain those terms.

The index builder speeds up queries by providing direct access to the requested data, referred to as index seek operation, rather than scanning the entire database for a few records. We use Elasticsearch,^12^ an open-source search and analytics engine, to create an index for our articles. The output from the index builder is a set of indexed articles that are ready to be stored in the reference-standard database.

#### Reference-standard database

We store the texts and metadata from the scientific articles that are already indexed in our reference-standard database. We refer to each piece of text (paragraph or full article) that is stored in this database as a ‘document’. We use Elasticsearch as the backend for this database. The output from this database goes into the QA pipeline.

#### Question answering pipeline

The QA pipeline searches through a large collection of documents from the reference-standard database for a span of text (paragraph or context) that answers a question. We choose to work with an extractive QA task [18] that can extract precise answers to questions posed in natural language from a given paragraph. This is because the precision of scientific language related to COVID-19 (such as information about genes, vaccines, mutations, chemicals) is critical in this work, so an extractive QA fits our goal. Unlike typical extractive QA models [17, 36, 53], in which end-users must explicitly provide a context, our QA pipeline finds the context (supporting paragraphs) for each question and extracts a short snippet as an answer. The QA pipeline consists of three sequential stages: (1) data retrieval; (2) data reading; and (3) answering.

*Data retrieval* The data retrieval phase is handled by the Retriever node. The Retriever receives a set of indexed documents as input and traverses the entire reference-standard database to find a set of candidate documents relevant to each question. It acts as a lightweight filter for locating the best candidate documents by calculating the similarity between the question and the documents. We use the Best Matching 25 (BM25) algorithm [54, 55] in the Retriever node to estimate the relevance of documents to a given question with possibly different degrees of importance, term relevance and sequence length. The BM25 is an advanced ranking function that is based on the Term Frequency-Inverse Document Frequency (TF-IDF) model [56]. The Retriever outputs a set of ranked documents that it deems most relevant to a query.

*Data reading* The data reading is handled by the Reader node that reads through documents’ texts in detail to find an answer. The input to the reader is a set of ranked documents returned by the Retriever. We use a Transformer-based model [28, 41] in the Reader node. In that, we fine-tune MPNet (a Transformer model) [30] to fit on our gold-standard dataset. More details about fine-tuning to MPNet are included in the next “Fine-tuning MPNet on the gold-standard dataset” section. The output from Reader is a list of answers for each question. An evaluation node is added after the Reader node to assess its performance.

*Answers* We chain both Retriever and Reader nodes together in the QA pipeline. This pipeline is represented as a directed acyclic graph of component nodes, which enables the creation of custom query flows, the merging of candidate documents for a Reader from the Retriever, and the re-ranking of candidate documents. The QA pipeline generates a ranked list of answers based on the question being asked. Each answer is accompanied by additional information, which is a context or paragraph from which the answer is extracted. Along with each answer, we show the model’s confidence (accuracy) in the extracted answer.

#### Fine-tuning MPNet on the gold-standard dataset

Transformer-based models like Google’s BERT [41], XLNet [57], and Facebook’s BART [58] have demonstrated outstanding results in a wide range of NLP tasks [59], such as question answering, classification and related language modelling tasks. These models are pre-trained on huge datasets and achieve a large-scale language understanding. We can further train these models on our dataset. This means, we can still use the pre-trained corpus, but we are adding a new layer on the top of the model and adapting it to our specific use case. This step is known as fine-tuning, as we fine-tune a large pre-trained Transformer model to fit our data.

**Fig. 4:**
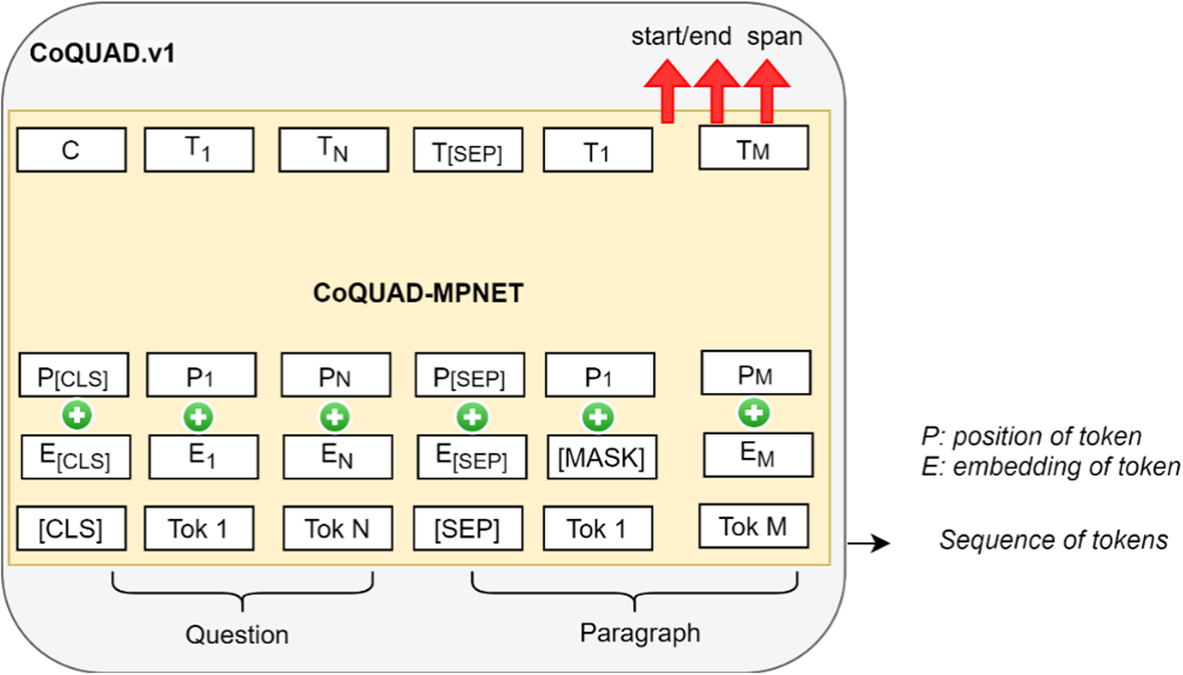
CoQUAD-MPNet

Recently, Microsoft introduced a Transformer-based model called MPNet (Masked and Permuted Pre-training for Language Understanding) [30], which inherits the benefits of both the Masked Language Modelling (MLM) of BERT [41] and the Permuted Language Modelling (PLM) task of XLNet [57], resulting in a stronger pre-trained model. In this work, we fine-tune the MPNet model on our gold-standard dataset, CoQUAD, and name it CoQUAD-MPNet. We release the weights of CoQUAD -MPNet here^13^ and show it in Fig. 4. We call this model CoQUAD.v1, because this is our first release of CoQUAD and we plan to update this dataset and model weights, based on more literature available in near future.

We use CoQUAD- MPNet inside the Reader module. The reason we add a Transformer model inside the Reader is to transfer the knowledge from powerful neural networks into our specialized task. The Reader module, with the help of CoQUAD-MPNet, is then again fine-tuned on our reference-standard data (CORD-19 and LitCOVID), which strengthens the working of our QA pipeline.

## Experimental setup

We evaluate our QA pipeline to determine how well it performs overall. We add a separate evaluation node after the Retriever module (shown in Fig. 3) that evaluates the Retriever for the returned documents. To evaluate Retriever, we check whether the documents returned by the Retriever contain the correct answers. We add a separate evaluation node after the Reader module that evaluates the answers returned by the reader. To assess the Reader, we see if the selected answer span in the document corresponds to the correct answer(s). We evaluate the QA pipeline using the gold labels (answers) from our gold-standard data. The gold label [50] is the ground truth value, which is an ideal predicted result based on humanly verifiable observation.

### Baseline approaches

We use the following baseline methods to compare against our model.

- **BERT** [41]: Bidirectional Encoder Representations from Transformers (BERT) is a Transformer-based model pre-trained using a combination of MLM objective and next-sentence prediction on Wikipedia and Toronto book corpora. In this work, we use the BERT-Base, Uncased, which has 12 layers (transformer blocks), 12 attention heads, and 110 million parameters.
- **XLNET** [57]: XLNet is a new unsupervised language representation learning method based on a novel generalized PLM objective. XLNet integrates ideas from Transformer-XL [60] model. In this work, we use XLNet-Base, Cased with 12-layers, 12-heads and 110 parameters.
- **ALBERT** [61]: A Lite BERT (ALBERT) is a version of BERT for language representation with much fewer parameters. We use ALBERT-Base having 12 repeating layers, 128 embedding layers, 12-heads and 11 million parameters.
- **BART** [58]: Bidirectional and Auto-Regressive Transformer (BART) is a Transformer-based model that uses a standard sequence-to-sequence architecture with a bidirectional encoder (like BERT) and a left-to-right decoder (GPT-2). In this work, we use the BART-Base model with 12 layers (6 encoder and decoder layers) and 217 million parameters.
- **ELECTRA** [62]: Efficiently Learning an Encoder that Classifies Token Replacements Accurately (ELECTRA) is a Transformer-based model like BERT that uses less computational power, smaller datasets, and less training time. In this work, we use Electra-small with 12 layers and 14 million parameters.
- **Funnel** [63]: A Funnel Transformer is a type of Transformer that gradually compresses the sequence of hidden states to make it shorter, lowering the computation cost. In this work, we use a Funnel-transformer-small version having 14 layers, 12-heads and 130 million parameters.
- **Longformer** [64]: Longformer is a modified Transformer that processes long sequences and scales quadratically with sequence length. In this work, we use Longformer-Base with 12-layer, 12-heads, and 149 million parameters.
- **COBERT** [22]: COVID-BERT (COBERT) is a retriever-reader system that answers the questions from the CORD-19. It is based on a TF-IDF vectorizer for retrieving documents and a fine-tuned DistilBERT model to read text.
- **COVID-QA** [29]: COVID-QA is a QA system based on the Robustly optimized BERT approach (RoBERTa) model [65].

We report the results for each baseline according to its optimal hyperparameter setting and report the best results for each baseline.

### Evaluation metrics

In this work, we make use of the following evaluation metrics:

- Precision, Recall, Mean Reciprocal Rank (MAR), Mean Average Precision (MAP) for Retriever
- Accuracy, F1-Score, Exact Match (EM) and SAS (Semantic Answer Similarity) for Reader.

Next, we explain the basic terms used for precision, recall, and accuracy in Table 2.

**Table 2:** Terms used for precision, recall, and accuracy

	Relevant	Non-relevant	Total
Retrieved	A	B	A + B
Not retrieved	C	D	C + D
Total	A + C	B + D	A + B + C + D

#### Evaluation metrics for retriever

We define the evaluation metrics that we use for Retriever here:

*Precision* is the fraction of retrieved documents that are relevant [35], as shown in Eq. (2):

2 $$\begin{array}{*{20}c} {Precision = \frac{A}{A + C} } \\ \end{array}$$

*Recall* is the fraction of relevant documents that are retrieved [35], as shown in Eq. (3):

3 $$\begin{array}{*{20}c} {Recall = \frac{A }{{A + B}}} \\ \end{array}$$

Both precision and recall are measures of goodness related to relevance [52].

*Mean Reciprocal Rank (MRR)* is a relative score that calculates the average of the inverse of the ranks at which the first relevant document is retrieved for a set of queries [35]. When the relevant ranks are averaged across the set of queries, this measure is called MRR. It is represented as shown in Eq. (4):

4 $$\begin{array}{*{20}c} {MRR = \frac{1}{\left| Q \right|}\mathop \sum \limits_{i = 1}^{\left| Q \right|} \frac{1}{{rank_{i} }}} \\ \end{array}$$

where $rank_{i}$ is the position of the relevant result in the *i*^th^ question and *Q* is the total number of questions.

*Mean average precision (MAP) is* the average precision across multiple queries [35]. Precision is calculated at each point when a new relevant document is retrieved. The average is then determined for each query, as shown in Eq. (5):

5 $$\begin{array}{*{20}c} {MAP = \frac{1}{\left| Q \right|}\mathop \sum \limits_{i = 1}^{\left| Q \right|} AP\left( q \right)} \\ \end{array}$$

where *Q* is the total number of questions and *AP(q)* is the average precision for a question*.*

#### Evaluation metrics for reader

We define the evaluation metrics that we use for the Reader here:

*F1 score*^14^ is a combined metric that incorporates both precision and recall by taking their harmonic mean. It can be represented as shown in Eq. (6):

6 $$\begin{array}{*{20}c} {F1 = 2*\frac{{\left( {precision} \right)*\left( {recall} \right)}}{precision + recall}} \\ \end{array}$$

*Exact match (EM)* measures the proportion of documents where the predicted answer is identical to the correct answer [31]. If the answer returned by the model perfectly matches the ground truth answer, we get a 100% EM score; if it does not, we get a lower score.

The EM and F1 score are two dominant metrics in the SQuAD evaluation.

*Accuracy* is defined as the proportion of correctly classified items, either as relevant or as irrelevant [35]. It is represented as shown in Eq. (7):

7 $$\begin{array}{*{20}c} {Accuracy = \frac{A + D}{{A + B + C + D}}} \\ \end{array}$$

*Semantic Answer Similarity (SAS)* [66] metric takes into account whether the meaning of a predicted answer is similar to the annotated gold answer, rather than just the exact words comparison as in other IR measures (F1 score, EM). We employ “cross-encoder/stsb-RoBERTa-large”,^15^ a Transformer model, to determine the semantic similarity of the two answers.

We show the results of all metrics during different values of top@ k. The top@ k refers corresponds to the number of relevant results among top-k retrieved documents. In this study, we employ *k* values of 1, 5, 10, and 20 based on common heuristics in IR evaluation [52]. All these metrics return a score in the range between 0 and 1. Usually, a higher score on these metrics is considered a better value.

### Hyperparameters

For training, we used an Nvidia Tesla P100 GPU with 16 GB RAM and 2 TB disk storage. We train the model in two steps: first to fine-tune the CoQUAD-MPNet, and then to train the actual QA pipeline. In both models, we have set the total batch size for training to 16. We set the ‘max query length’ as 64 tokens, which is the length of the question’s input tokens; anything longer is truncated. We set the ‘Document stride’ to 128, which is the size of the stride when splitting documents into chunks. We set the ‘Max sequence length’ to 512 (its default value), which is the length of the input document sequence. We set the ‘max answer length’ to 50, which denotes the maximum size of the answer that can be generated. Finally, the Adam [67] weight decay is used as optimization with a learning rate of 1*e*-5. All the other hyperparameters are set to their optimal values. Furthermore, the Reader is fine-tuned using an annotated dataset in the same format as the SQuAD dataset. Each experiment is repeated at least 10 times. All the baseline models are also optimized to their optimal settings, and we report the best result for each model.

## Results

In this section, we evaluate the quality of the results of our CoQUAD system. We perform the experiments on our reference-standard dataset. The Reader module of CoQUAD is evaluated based on the gold-standard dataset. The goal of this evaluation is to see how well our model works in each setting and which module of the pipeline needs to be improved.

### Evaluation of CoQUAD QA pipeline

We evaluate both the Retriever and Reader modules individually to test the performance of the whole QA pipeline. Each module is evaluated based on its evaluation metrics. The results are shown in Table 3.

**Table 3:** Evaluation of QA pipeline

Evaluation metric	Top@ 1	Top@ 5	Top@ 10	Top@ 20
*Retriever*				
Recall (single document)	0.495	0.711	0.720	**0.836**
Recall (multiple documents)	0.494	0.716	0.720	**0.836**
Mean reciprocal rank (MRR)	0.495	0.572	0.582	**0.775**
Precision	**0.495**	0.344	0.342	0.304
Mean average precision (MAP)	0.494	0.672	0.690	**0.697**
*Reader*				
F1-Score	0.504	0.636	0.636	**0.771**
Exact match (EM)	0.539	0.549	0.698	**0.775**
Semantic answer similarity (SAS)	0.503	0.623	0.687	**0.785**
Accuracy	0.895 (same for all top @k)			

Bold means best result

#### Performance of the retriever in the pipeline

The result in Table 3 shows that as we increase the value of top@ k, the recall, MRR, and MAP scores of the Retriever improve (get higher close to 1). We also see that as we increase the value of top@ k, the recall improves for both single and multiple documents. This is demonstrated by quite high recall scores during the top@ 5, 10, and 20. We get 83.6% recall during the top@ 20 for both single and multiple documents. A higher value of recall shows that our system can retrieve many of the truly relevant documents in response to each question.

Normally, when the recall increases, the precision drops [52, 68] and vice versa. The precision shows the number of relevant items that are returned. In these results (Table 3), we see that the precision of Retriever decreases as top@ k increases, and the overall precision score is lower when compared to the recall scores. For us, recall is more important for the Retriever, which we explain with an example below.

Assume there are five documents: D1, D2, D3, D4, and D5 in the database and only three documents: D3, D4, and D5 are relevant to the query: “What is COVID-19?”. If the model returns the documents D2 and D3, of which only D3 is relevant, then the number of documents that are *retrieved and relevant* is 1 (only D3). As a result of the formula, recall equals 1/3 = 0.67 (1 is relevant and retrieved, and 3 is the number of relevant documents) and precision equals 1/2 = 0.5 (1 is relevant and retrieved, and 2 is the total number of returned documents). While the precision score is higher in this specific example, it does not accurately reflect a model’s overall performance. In our study, we are more interested in determining the total number of relevant documents that are retrieved, so recall is a higher priority for our system.

Due to this tradeoff between precision and recall, we also show the performance of our model using MAP that combines both recall and precision for ranked retrieval results. MAP shows the mean of the precision scores after each relevant document is retrieved. The MAP score of our Retriever is also high (around 70% during top @20). A higher MAP score indicates the average precision per retrieved list and thus the order of the documents in a list. A good MAP score means that the recommended list contains many relevant items.

We show the Retriever’s performance for the MRR score, which also increases with increasing top@ k, reaching 77.5% during top @20. This indicates that our method is more than 77% accurate at focusing on the first relevant element in the list. This is typically more appropriate for targeted searches, such as those in which users inquire about the first best item.

#### Performance of reader in the pipeline

We evaluate the performance of Reader for the accuracy, F1-Score, EM and SAS scores. The Reader is evaluated based on how well it extracts the best answers from the documents retrieved by the Retriever. The results in Table 3 show that our Reader module is accurate in returning us the correct answers, with an accuracy score of approximately 90%.

We also evaluate Reader’s ability to give a precise answer through the EM ratio, which is around 54% during top @ 1, increases to about 70% during top @ 10, and to 77.5% during top @ 20.

With an increase in top@ k, the Reader’s F1 score improves as well. In these experiments, the Reader’s F1 score is around 77% during top@ 20. This F1-score in Reader is calculated by comparing the predictions to the actual answer. This score is determined by the number of shared words between the prediction and the truth, where precision is the ratio of shared words to total words in the prediction and recall is the ratio of shared words to total words in the ground truth.

Normally, for the SQuAD task, the SAS is an important metric [31] that measures the semantic similarity between different words and doesn’t penalize the model for ignoring the lexical overlap. The SAS score of our Reader is around 50% during top @1 and rises to 78.5% at the top @20. This demonstrates the high semantic textual similarity between the predicted and the ground truth answer. The SAS and F1-score in a Reader can be explained with an example below:

- *Question:* “What are the organs affected by COVID‐19?”.
- *Ground-truth answer (actual answer):* “Lungs, kidneys, brain, and liver are the organs affected by the COVID-19”.
- *Predicted answer:* “Lungs are most affected”.

In this example, we obtain an F1 score of 0.5 and a SAS score of 0.69. The discrepancy between these scores is explained as follows: precision is equal to 1 (all tokens or words in the predicted answer are included in the ground-truth answer), but recall is less than one (not all ground-truth tokens are included in the predicted answer), lowering the recall score. So, we obtain an F1-score of 0.5 (harmonic mean of precision and recall scores). SAS, on the other hand, awards the answer a near 0.7 score, indicating that it correctly captures the semantics in the majority of the tokens.

**Fig. 5:**
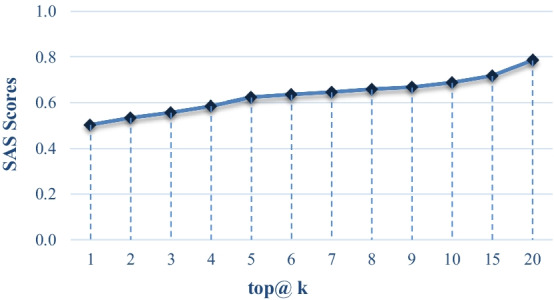
SAS scores of Reader during different values of top @k

We also show the Reader’s effectiveness on different numbers of retrieved answers using SAS scores in Fig. 5. In this experiment, we retrieve 100 documents using Retriever and then specify the top@ k (k is ranging from 1 to 20) answers to be retrieved from the candidate documents.

The result in Fig. 5 shows that our CoQUAD system gets credit for a correctly-identified answer in any retrieved document. This means our system is demonstrating higher relevancy when we specify a higher value of top@ k. We chose top@ 20 as the maximum range for top@ k because, in most cases, a user will only wait for a limited list of top-k recommendations before seeing the best results.

Finally, we see in Table 3 that the accuracy of the Reader is around 90%. Since we get the same accuracy across different values of top@ k, we mention it once in Table 3. This accuracy score shows the overall performance of our Reader module to identify the correct results from all the documents.

### Comparison with baselines

We also evaluate the performance of various QA systems to our CoQUAD system. The primary goal of this set of experiments is to determine how well various QA systems perform. Because the task is to evaluate the answers returned by a QA model, we evaluate these models using the EM and F1-score, while adhering to the SQuAD evaluation standard [31].

**Fig. 6:**
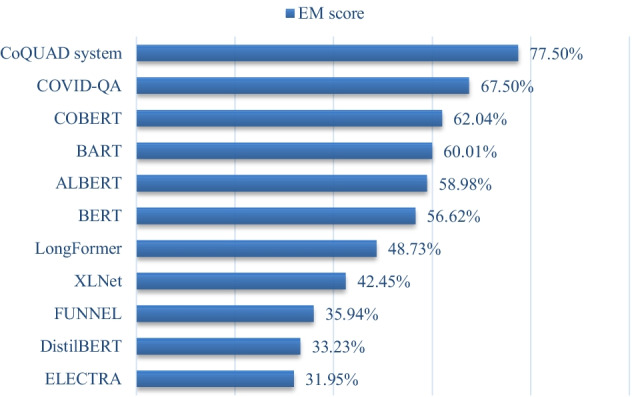
EM score of all models

**Fig. 7:**
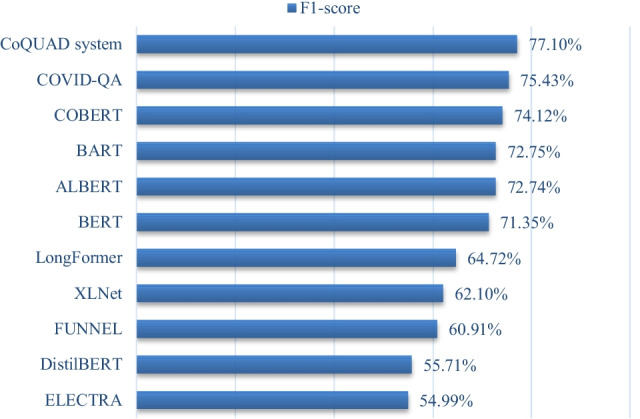
F1-score of all models

The EM and F1 scores are also used to assess the Reader module in the whole pipeline. We show the comparison of our model and baseline methods for the EM score in Fig. 6. We also show the performance comparison of all methods USING F1-score in Fig. 7.

As shown in Figs. 6 and 7, our QA model outperforms all baseline models for the EM and F1-score. This is demonstrated by our model’s highest EM score, 77.50%, and highest F1 score, 77.10%. The superiority of our CoQUAD system is attributed to our fine-tuned MPNet model that we use inside the Reader module. The MPNet outperforms the standard MLM task of BERT and the PLM task of XLNET [30] and achieves better results on this QA task. MPNet also leverages both the MLM and PLM tasks by rearranging and segmenting the tokens in the input sequence.

Next comes the performance of COVID-QA [29] which is based on RoBERTa [65]. RoBERTa has also shown good performance on the SQuAD task [65]. It has demonstrated an EM score of 67.50% and a F1 score of 75.43%. The original COVID-QA model uses CORD-19 data, but the version of the data used in COVID-QA is not very recent. In contrast, we include both the older and newer models of the CORD-19 version in this work.

The overall performance of COBERT [22] is also competitive, coming in second place to COVID-QA, it has an EM score is 62.04% and F1-score is 74.12%. The COBERT architecture is also based on a retriever and reader architecture, but unlike our model, the COBERT architecture makes use of standard techniques, such as TF-IDF in the retriever and a fine-tuned DistilBERT in the reader. The advantage of our approach is that we employ more sophisticated techniques such as BM25 in the retriever node to estimate the relevance of documents to a given question. Additionally, we use MPNet, which works better than the Bert (or DistilBERT) and XLNet models [30], as it combines both the MLM and PLM tasks.

Then comes the performance of BART, ALBERT, BERT, LongFormer, XLNET, FUNNEL, DistilBERT and ELECTRA in the same order. BART has given us an EM score of around 60% and an F1 score of ~ 73%. We feed the entire document into the encoder and decoder to use BART for QA tasks. BART also performs well in SQuAD and can handle sequences with up to 1024 tokens [58]. BART has also worked well as a baseline in this experiment, implying that it could be a good candidate model for the Reader module. the ALBERT model has been ranked among the very best models for QA tasks on a variety of datasets, including the SQuAD 2.0 dataset [69]. We also find that in our experiments, the ALBERT model performs better than the original BERT.

Then comes the performance of BERT, Longformer and XLNET. The XLNet model introduces PLM, which predicts all tokens in random order. This is in contrast to BERT’s MLM task, which predicts only the masked (15%) tokens. Both models have shown medium-level performance in our experiments. However, when we use MPNet, we take the advantage of both the models, that’s why we see better performance with MPNet in our work.

The Longformer performs at a medium level in this experiment. One advantage of Longformer compared to BERT is that it can handle longer sequences of text. For example, the text of a scientific article normally consists of 5000 words or up, Longformer can handle these long sequences. However, in our work, BERT has shown better performance than Longformer. This is probably because adjusting long sequences is not an issue in our model, as we can fit all the data into the memory by using the proper batch sizes and by adjusting various hyperparameters (document stride question length, sequence length).

Then, comes the performance of Funnel, DistilBERT and ELECTRA, all of which are different versions of BERT and have demonstrated some performance in the SQuAD tasks. These models are normally more useful in scenarios where memory utilization is an issue. One can benefit from these models if the goal is to get a BERT-level performance with limited resources (memory, disk, CPU cycles).

Our CoQUAD outperforms all these baseline models in EM and F1-score. For the other QA systems, we need to give explicit contexts, however, our CoQUAD does not require any explicit contexts. it can derive the contexts from a large pool of documents that are used to train the QA pipeline.

We address our second research question: “How to find the answer(s) to a given question from a large set of documents” through these above experiments (performance of Retriever and Reader in the pipeline). These experiments demonstrate that our proposed solution is capable of retrieving precise answers from a pool of documents with a high degree of accuracy and correctness.

## Discussion

### Practical impact

The findings of this study have several theoretical and practical implications. The CoQUAD system can be used to answer questions about COVID-19 from the scientific literature, as well as to investigate COVID-19 unintended consequences.

*Reference-standard dataset:* We create a reference-standard dataset from the COVID-19 literature by scientifically parsing the articles from CORD-19 and LitCOVID initiatives. We explain the data construction steps in detail to make it easier for researchers, working along this line of research, to follow the steps and build on such datasets, as well as to analyze and synthesize information from a large quantity of content.

*Gold-standard dataset*: To the best of our knowledge, there is no gold-standard dataset and a model to-date that also focuses on the post-COVID-19 condition. We explain, in this work, how to build and annotate a gold-standard dataset to facilitate research in this field. Thus, our system and dataset may be useful for a QA task involving COVID-19 literature. Obtaining a gold standard dataset for the evaluation of a QA system is also an expensive and time-consuming task. We prepare this data that can be used to evaluate other COVID-19 QA systems also. This dataset can also be used to make models like the Reader module in this work, either as a standalone model or a part of a larger system.

By constructing these two datasets (reference-standard and gold-standard), we also answer our first research question (How to construct a dataset to find evidence from scientific literature?) in this work.

*A question-answering system*: The purpose of this research is to develop an AI-driven QA system for mining scientific literature. The proposed system addresses the shortcomings of current biomedical portals, such as the requirement for proximity searches on the PubMed interface using Boolean conjunctions “AND” or “OR”, or using phrasal searches, which complicates the process and increases the likelihood of missing relevant articles. When our approach is used, users are only required to enter a query in natural language; the CoQUAD system handles all intermediate operations. When properly implemented, such a system can assist both information specialists and practitioners in their search for biomedical or other scientific literature. As a result, it is recommended that researchers begin with one of those databases (such as CORD-19, LitCOVID or alike) when using an interface to develop and fine-tune retrieving and reading strategies. By designing this QA system, we thereby address our second research question (“How to find the answer(s) to a given question in a large collection of documents?”).

*CoQUAD-MPNet:* The CoQUAD-MPNet model is based on the Transfer learning paradigm, so, it can be applied to a variety of downstream tasks such as summarization, text classification, and translation with minimal additional task-specific training.

*Adaptability of CoQUAD to various public health issues:* We have designed the CoQUAD system to be adaptable and reusable. Each phase of this workflow can be tailored to other related use cases, involving either further COVID-19 research or other emerging public health issues.

The methodology of CoQUAD is composed of three primary components: data collection, data processing pipeline, and QA pipeline. Each component contributes to complete adoption to other domains, minimizing the amount of information transferred from the initial training to the adaptation process. Our pipelines can also be easily adapted for repeated processing of a dataset with minor parameter changes or for processing multiple datasets, saving a significant amount of developers’ force and time.

We are currently using COVID-19 literature data, but the same architecture can be re-used to provide QA for other health science topics based on the related literature. The only requirement is that the data collection phase be altered. Following a change in data sources, the data processing pipeline will remain unchanged. Because the QA pipeline is also built using transfer learning techniques, it can be easily fine-tuned to the other related task (classification, clustering, summarization, translation, predictions or so) at hand.

The following are a few examples of how this architecture might be used for other emerging public health issues:

- To study the risk factors related to other diseases (e.g., cancer, diabetes, cardiac and so) and disease management.
- To investigate the research about clinical drugs aimed at evaluating a medical, surgical, or behavioural intervention.
- To analyze the impact of non-pharmaceutical interventions (actions that people and communities can take to help slow the spread of illnesses) on population groups
- To study the public health conditions that are generally linked to other measures of social vulnerability such as low socioeconomic status and poor quality of housing
- To provide answers related to health disparities and suggest health equity
- To provide information related to the transmission of disease that may be symptomatic and asymptomatic in patients
- To link to several studies related to forecasting and modelling.

As mentioned earlier, the only requirement for a new task is the change in the data source. For example, we can use many of the BioASQ^16^ datasets, which are also in SQuAD format as input to this pipeline. However, the SQuAD format of the input data to the CoQUAD architecture is not a requirement, it can take any text data and parse it for the retrieval and reading tasks. For the state-of-the-art QA systems, providing a SQuAD format of input data is required [31]. The only time when we need the SQuAD format is when we need to fine-tune or evaluate the Transformer model in the Reader module. This is because the Transformer model like MPNet is an outside model. There are a few criteria for reproducibility and adaptability of CoQUAD architecture:

- The data and metadata for the new use case (e.g., risk analysis, drugs exploration, clinical task and so) must be provided to the data processing pipeline to adopt the new task.
- The dataset to evaluate the Reader component for the human level performance should be in the SQuAD format.
- If the goal is to change the Transformer model embedded in the Reader module, the hyperparameters, number of layers, attention heads, and model weights must be specified; if the same Transformer model is to be used, no changes are required.

As with any other system, the individual steps involved in a pipeline design should be analysed upon changing the data source. Without monitoring, it is impossible to determine whether or not the system is performing as expected.

We show the demonstration of CoQUAD in Additional file 1: “Appendix B”: Use case. Next, we discuss some of the uses, limitations, and future directions of CoQUAD.

### Limitations and future directions

*A closed-domain, extractive QA system:* At the moment, our CoQUAD system is a closed-domain, extractive system. This means it can only provide answers within its domain if the answer can be found in the literature. While this is a limitation, it is also a benefit. This is because given the evolving and changing evidence on the SARS-CoV-2 and its variants, as well as the sensitivity of the biomedical domain, we cannot rely on an AI system to generate accurate answers based on its intelligence. In future, we may want to try to develop a QA system based on Knowledge Graphs [70], once our model is more mature and able to extract new information using different knowledgebases. A knowledge graph represents a network of real-world entities (objects, events, situations, or concepts) and demonstrates the underlying relationship between these entities.

*Critical appraisal*: So far, there is no mechanism for critical appraisal in this work. Critical appraisal [71, 72] is the process of thoroughly and methodically examining research to determine its trustworthiness, as well as its value and relevance in a given context. This is a common limitation of deep neural network-based QA systems. One likely reason for this is that it is impractical to critically evaluate large amounts of data and streams of information used to feed deep neural networks in such QA systems. While this is a limitation, for now, research may be undertaken on having a critical appraisal in deep neural networks, especially in the sub-domain of transfer learning.

It is also important that we study the risk of bias assessment (sometimes referred to as quality assessment or critical evaluation) to ensure that evidence synthesis results and findings are transparent and accessible. Our goal is to contribute to high-quality science that improves public health and safety, a discussion of bias is critical for the journal's readers.

*Multilingual:* This study only includes studies published in English. While this requirement was necessary to ensure that the chosen studies were understandable to the authors. In the future, we would like to include more multilingual literature on this subject (COVID-19), as well as assess the methodical soundness of the studies included in this QA system.

## Conclusion

In this paper, we propose CoQUAD system to address COVID-19 challenges, assisting researchers and clinical workers and public in obtaining authentic scientific information in the form of QA. We build two datasets: (1) a reference-standard dataset consisting of scientific papers from the CORD-19 and LitCOVID initiatives and (2) a gold-standard dataset that is prepared and annotated by experts. CoQUAD consists of a Retriever that retrieves documents from the document store in response to a question; and a Reader built upon MPNet (a Transformer model) that extracts the specific answer to each query from the documents returned by the Retriever. The returned responses are then ranked and evaluated for exact word and semantic similarity. We evaluate our model using the gold-standard dataset. The experimental results demonstrate our model’s superiority over state-of-the-art models. This is due to the unique design of our model and the large amount of data collected in this research. The gold-standard dataset, in this work, is also used to enhance the Reader’s functionality. The results show the superiority of CoQuad against the baseline methods in extracting answers related to any COVID-19 questions from the literature. Our objective is to enable anyone to ask a COVID-19 related question in natural language and receive an up-to-date, accurate and specific response without having to review source documents or reviews. With this project, we hope to assist the medical community in developing answers to high-priority scientific questions, as well as in sifting through scientific articles more quickly and efficiently.

## Supplementary Information

**Additional file 1**.** Appendix A**. Exploratory analysis. This is basically the analysis of the reference-standard dataset.

## Data Availability

The data and methods can be made available upon request from the corresponding author on reasonable request.

## Abbreviations

WHO: World Health Organization
PHAC: Public Health Agency of Canada
IR: Information retrieval
NLP: Natural language processing
AI: Artificial intelligence
CoQUAD: COVID-19 question-answering dataset
QA: Question-answering
SQuAD: Stanford question answering dataset
CORD-19: Covid-19 open research dataset
RoBERTa: Robustly optimized BERT pretraining approach
GPT-2: Generative pre-trained transformer 2
BERT: Bidirectional encoder representations from transformers
COVID-QA: COVID-19 question answering
TF-IDF: Term frequency–inverse document frequency
BM25: Best matching 25
MPNet: Masked and permuted pre-training for language understanding
PHO: Public Health Ontario
MLM: Masked language modeling
PLM: Permuted language modelling
PDF: Portable document format
XML: Extensible markup language
COBERT: COVID-BERT
PMC: PubMed central
JSON: JavaScript object notation
PMID: PubMed unique identifier
ELECTRA: Efficiently learning an encoder that classifies token replacements accurately
ALBERT: A lite BERT
BART: Bidirectional and auto-regressive transformer

## Footnotes

1 http://www.worldometers.info/coronavirus.

2 https://www.canada.ca/en/public-health.html.

3 https://rajpurkar.github.io/SQuAD-explorer/.

4 https://ir.nist.gov/covidSubmit/data.html.

5 https://ai2-semanticscholar-cord-19.s3-us-west-2.amazonaws.com/historical_releases/cord-19_2021-12-20.tar.gz.

6 https://ftp.ncbi.nlm.nih.gov/pub/lu/LitCovid/litcovid2BioCXML.gz.

7 https://www.ncbi.nlm.nih.gov/pmc/about/intro/.

8 https://ftp.ncbi.nlm.nih.gov/pub/lu/LitCovid/.

9 https://pubmed.ncbi.nlm.nih.gov/.

10 https://annotate.deepset.ai/.

11 https://tika.apache.org/.

12 https://www.elastic.co/.

13 https://huggingface.co/shaina/covid_qa_mpnet.

14 https://scikit-learn.org/stable/modules/generated/sklearn.metrics.f1_score.html.

15 https://huggingface.co/cross-encoder/stsb-RoBERTa-large.

16 http://www.bioasq.org/.
