# Supplementary material for: CoQUAD: a COVID-19 question answering dataset system, facilitating research, benchmarking, and practice
Source: BMC Bioinformatics. 2022 Jun 2;23:210. doi: 10.1186/s12859-022-04751-6 (PMC9160513; doi:10.1186/s12859-022-04751-6)
Supplement: Supplementary file 1 — Additional file 1. Appendix A. Exploratory analysis. This is basically the analysis of the reference-standard dataset. [file 12859_2022_4751_MOESM1_ESM.docx]

Appendices

Appendix A: Exploratory analysis

### Preprocessing and analysis of CORD-19.

We performed an exploratory analysis on the CORD-19 dataset and show some of the analysis below. Figure S1 shows a word cloud (graphical representation) of significant words used in titles of CORD-19 articles, ranked by their frequency and relevance.


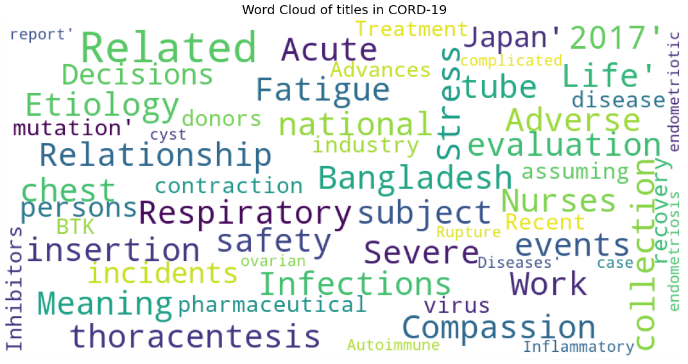


**Figure. S1.** Word cloud of titles in CORD-19

Figure S2 shows the word cloud of abstracts in CORD-19 data. Some of the important words that we see in both figures are: 'Acute',' Fatigue', 'Infections, 'events',' Respiratory', 'disease', 'etiology', which are commonly used in these COVID-19 papers.


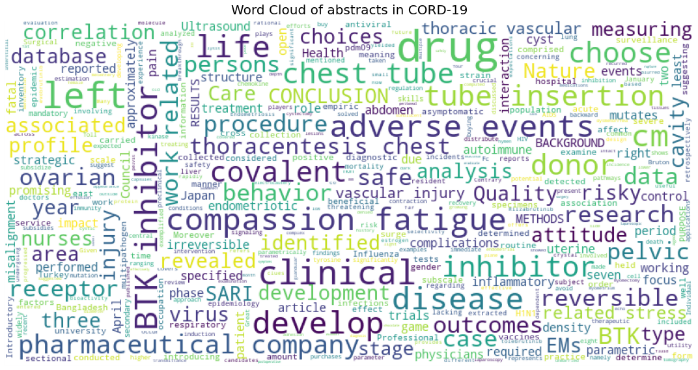


**Figure S2.** Word cloud of abstracts in CORD-19

Figure S3 below shows the important topics and the words inside each topic in the CORD-19. These topics are generated using BERTopic^[[1]](#endnote-1)^, a topic modeling technique that leverages BERT embeddings (73) and TF-IDF (52) scores to create dense clusters. Each topic number (topic 0, 1 and so) comprises of words that are pertinent to that particular topic. To generate a topic representation, we take the top-8 words from each topic (y-axis shows the words) ranked by their TF-IDF scores (scores are shown in the x-axis). TF-IDF is a statistical measure that measures how relevant a word is to a document in a corpus. Some prominent topics in Figure 3 are ‘sars’,’vaccine’-related, ‘pandemic’, ‘cardiac’ and so. A higher TF-IDF score shows higher information density that means a word with higher score is more representative of its topic.


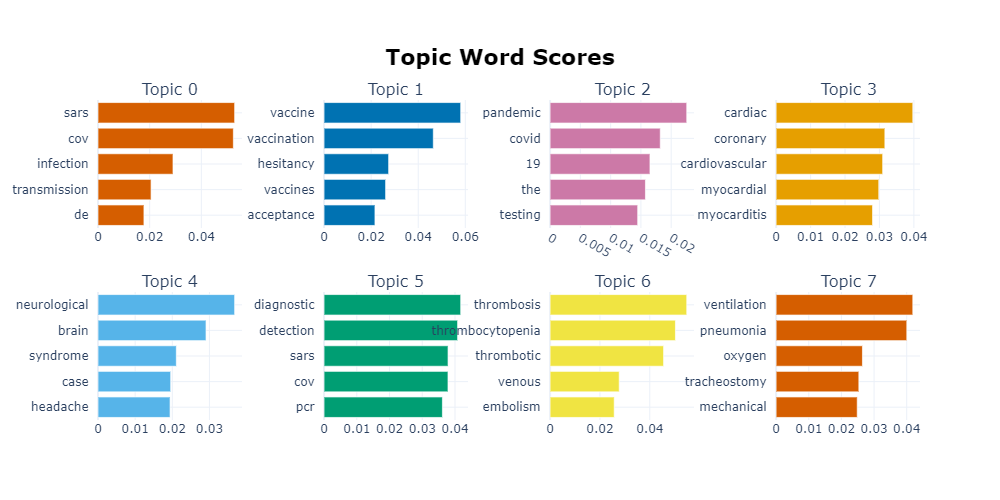


**Figure. S3.** Important topics and words used in CORD-19 papers

We also show the hierarchical relationships between different sets of topics. The x-axis in the hierarchical clustering shows the cosine distance among different topics. A higher cosine similarity score indicates that the topics are closer to each other and are more similar. We choose the top-20 topics in Figure S4 to show this clustering. Some of the interconnected and important topics, as seen in Figure S3, are related to ‘vaccine’, ‘sars’, ‘mental’, ‘cardiac’ and so.


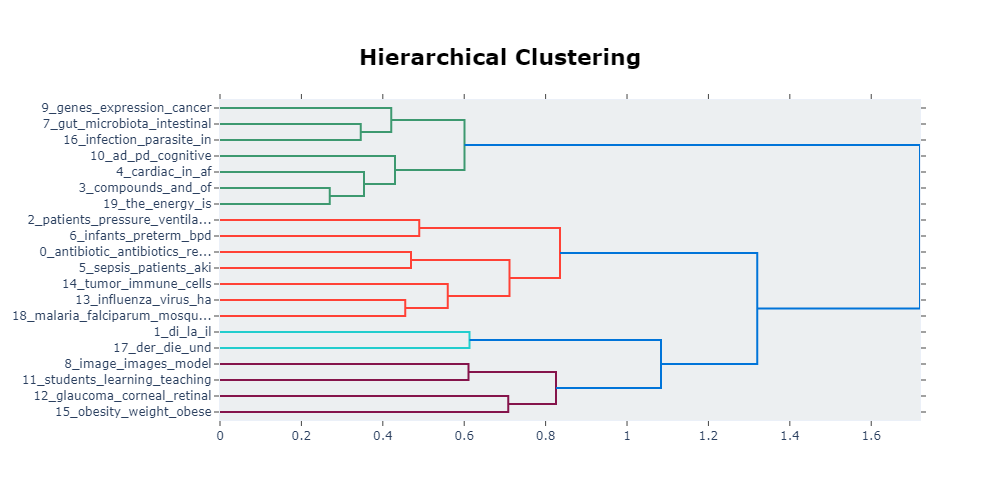


**Figure. S4.** Hierarchical clustering of top-20 topics in CORD-19 papers

We also show a similarity matrix of 5 clusters of documents with top-100 topics in Figure S5, where similarity is based how close a topic is with the other topics in a cluster. The scores on the right y-axis shows the similarity score between any two topics. These similarity scores are based on TF-IDF scores, where a higher value means topics are more similar to each other. This full matrix indicates how similar certain topics are related to each other. For example, we see the similarity of topic social media and suicide in school, in Figure S6, is 0.7121018, which is quite high.


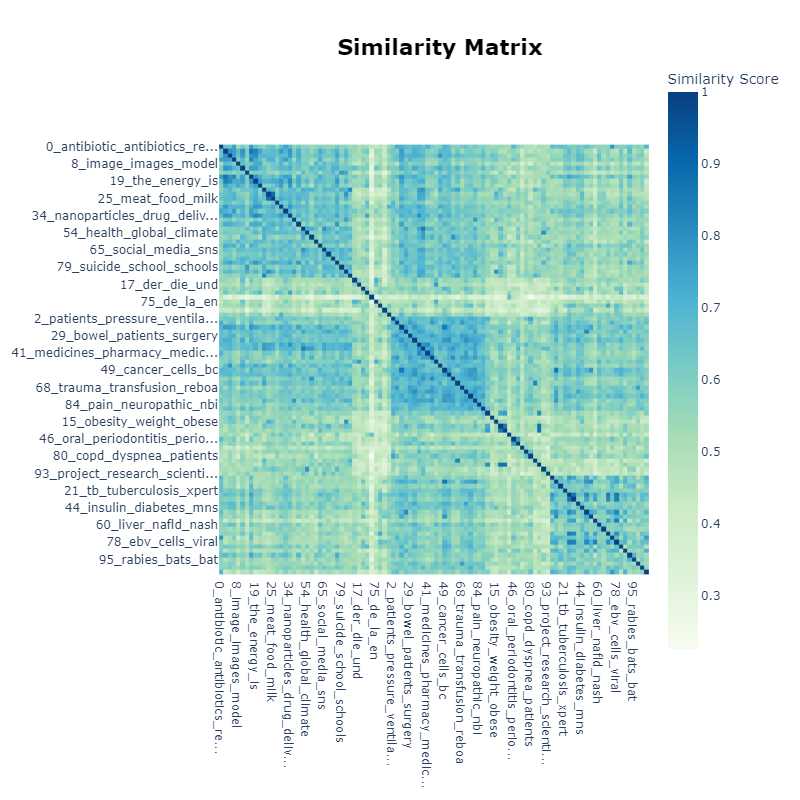


**Figure. S5.** Similarity matrix of topics using 5 clusters and 100 topics on CORD-19 dataset


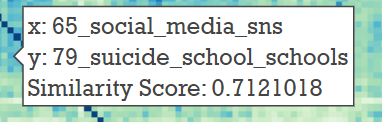


**Figure. S6.** Similarity score of two topics in the similarity matrix

### Preprocessing and analysis of LitCOVID

Figure S7 shows a word cloud in the titles of LitCOVID articles.


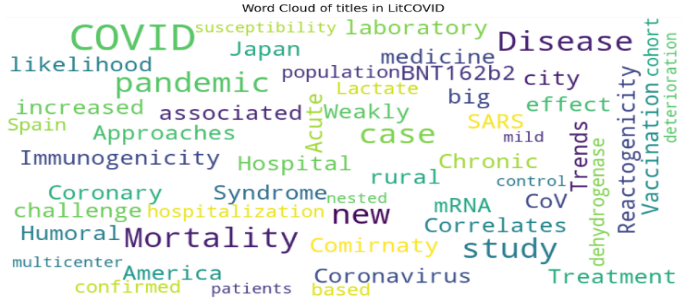


**Figure. S7.** Word cloud of titles in LitCOVID

Figure S8 shows the word cloud of abstracts. Some of the important words that we see in both figures are: 'COVID', ‘vaccine’, ‘Mortality, 'Disease’, 'events',' Respiratory', 'disease', which are commonly used in COVID-19 literature.


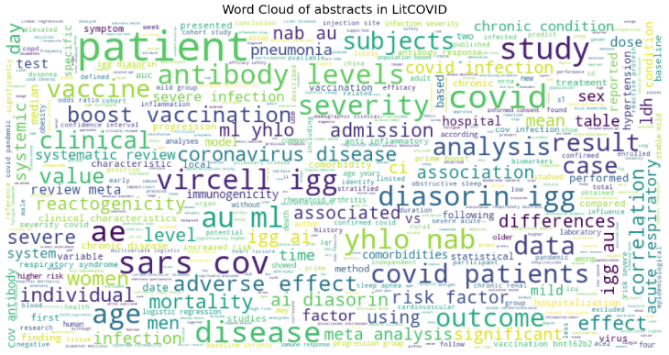


**Figure. S8.** Word count of abstracts in LitCOVID

Figure S9 shows the top-8 important topics and the words inside each topic, in the LitCOVID. We can see that some of the important topics in LitCOVID are ‘cardiac’, ‘samples’, ‘telehealth’, ‘mpro’, ‘chest’ and so.


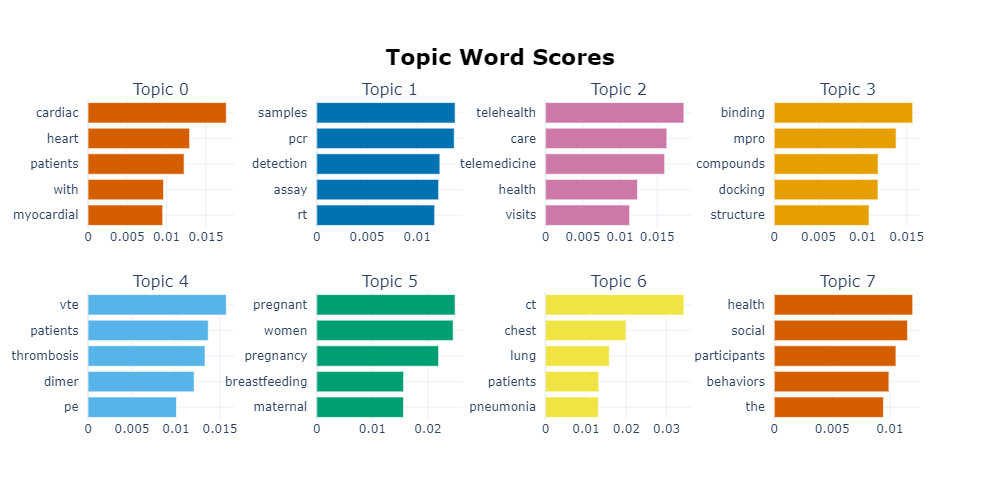


**Figure. S9.** Important topics and words used in CORD-19 papers

Figure S10 shows the hierarchical relationships between different sets of topics in the LitCOVID dataset. We can see that some of the interconnected topics in LitCOVID are ‘telehealth’, ‘cardiac’, ‘chest’, ‘pregnant’ and so.


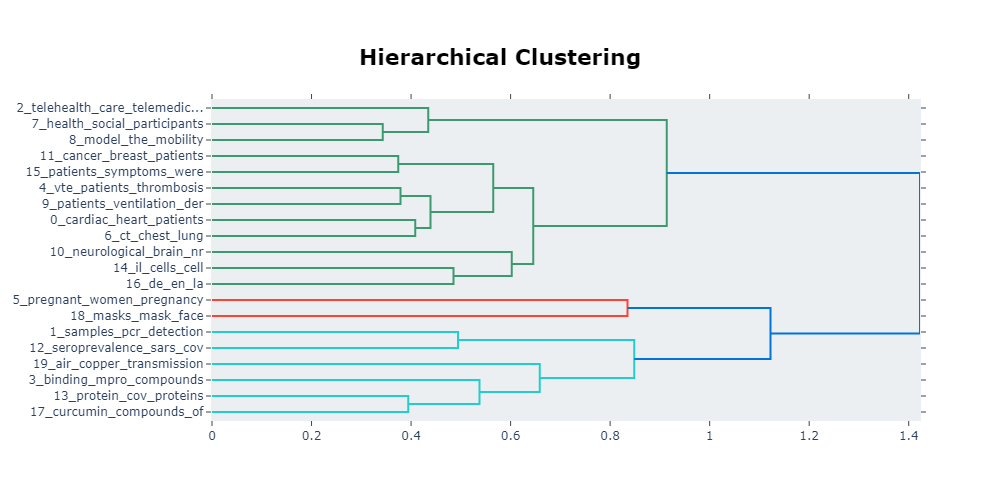


**Figure. S10.** Hierarchical clustering of top-20 topics in LitCOVID papers


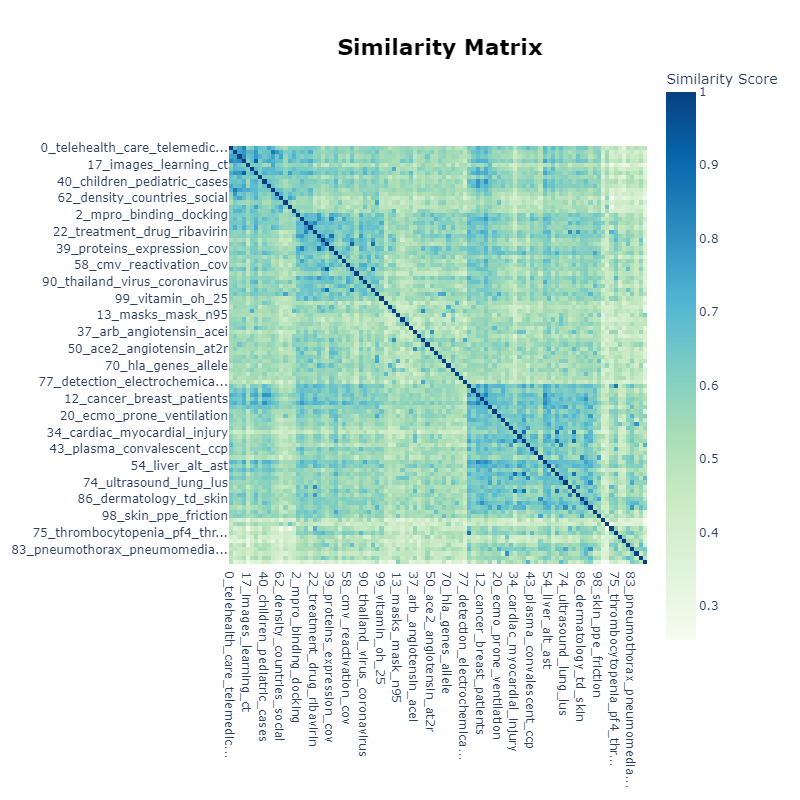


**Figure. S11.** Similarity matrix of topics using 5 clusters and 100 topics on LitCOVID dataset

We also show a similarity matrix of 5 clusters of documents with top-100 topics in Figure S11 and similarity of topic ‘tocilizumab’ and ‘antibody’ is 0.6082 in Figure S12.


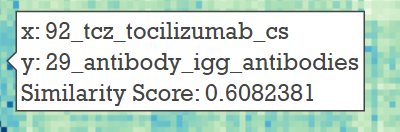


**Figure. S12.** Similarity score of two topics in the similarity matrix

Appendix B: Use case

Demonstration: We visually explore the effectiveness of CoQUAD approach. As a test set, we provide a collection of COVID-19-related publications that focus on the impacts of COVID-19 on marginalized communities. As an example, we first provide a question “What groups of people are more vulnerable towards the coronavirus?”, we specify top@ 2, which means we are interested in top-2 retrieved documents and ranked answers. The answers returned by CoQUAD system for this question are shown in Figure S13.


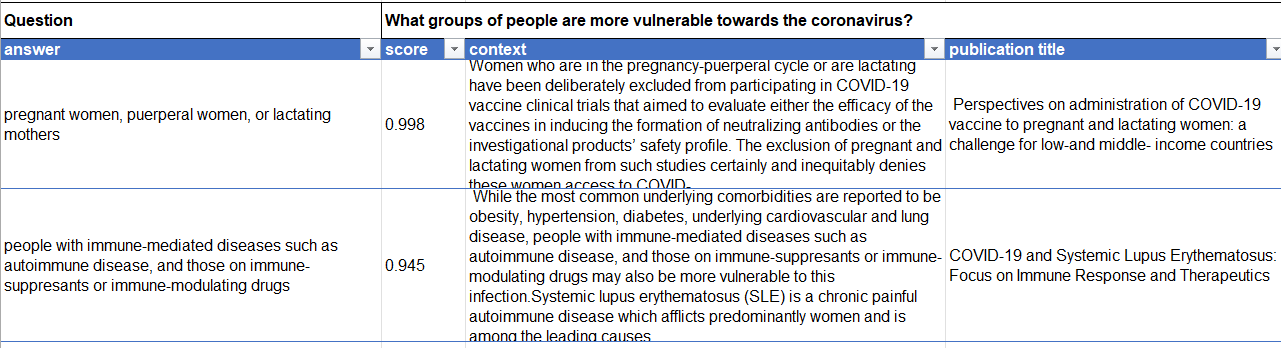


**Figure. S13.** Answers returned by CoQUAD during top@ 2.

Inside the CoQUAD, the Retriever performs an initial filter of the documents to find those documents that may contain answers to this question. The top-2 best matching documents from the Retriever are then passed to the Reader. The Reader examines the documents returned by the Retriever and extracts the best answers. We get these answers: “pregnant women, puerperal women, or lactating mothers” and “people with immune-mediated diseases such as autoimmune disease, and those on immune-suppresants or immune-modulating drugs” from two candidate documents, ranked by Reader. Our experts manually evaluate these answers and find the answers returned are quite accurate. We also input more questions, some of which are shown next.


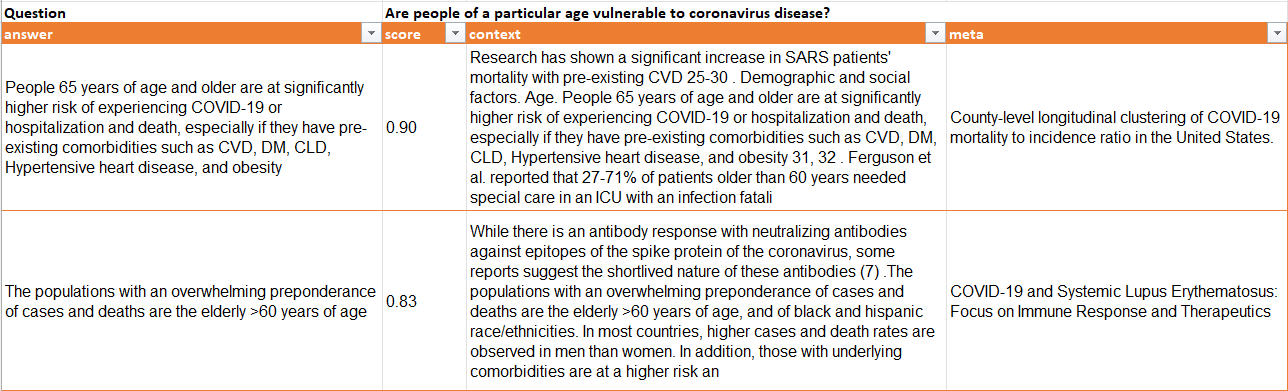


**Figure. S14.** Demonstration for “Are people of a particular age vulnerable to coronavirus disease?”

As shown in Figure S14, the answers to the question “Are people of a particular age vulnerable to coronavirus disease?” shows that people over 60 or 65 are more vulnerable to the COVID-19, which is a known fact and also validated in evidence-based research (74,75). We also input the question “Who are at risk of severe disease due to the coronavirus disease?” and find answers quite matching to what we are expecting.


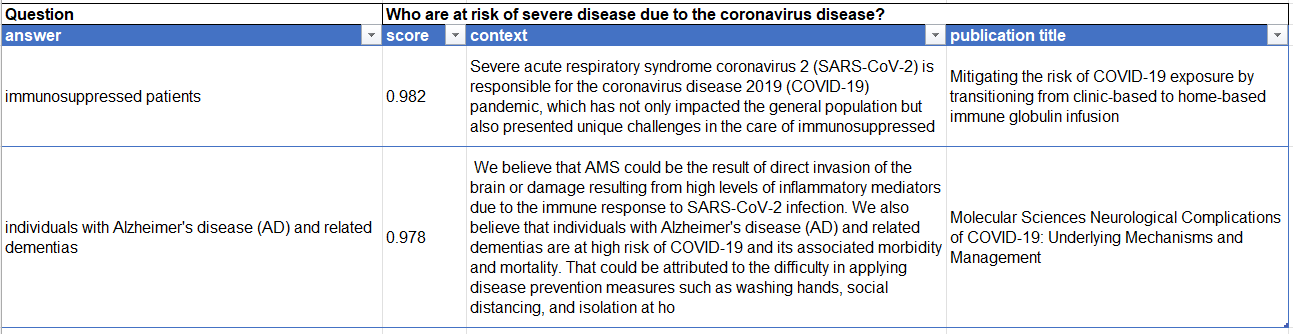


**Figure. S15.** Demonstration of “Who are at risk of severe disease due to the coronavirus disease?”

We input many more questions to the CoQUAD (other than shown here) and are satisfied with its overall level of performance when it comes to answering questions about COVID-19. CoQUAD also demonstrates the model's high confidence in extracting the correct answers, which is greater than 90% in these results (Figure S15). Each answer is also accompanied by a context, which is a surrounding paragraph from which the answer is extracted, as well as the title of the publication. The CoQUAD is designed such that it can also return more metadata information, such as the start and end of paragraphs and answers within the document, DOI, and URL, However, we only show limited and important information in these figures for the sake of clarity. Overall, our own examination of these answers reveals that CoQUAD achieves accuracy comparable to that of a human.

1. https://maartengr.github.io/BERTopic/api/bertopic.html [↑](#endnote-ref-1)
